# Efficacy and Safety of Resveratrol Supplements on Blood Lipid and Blood Glucose Control in Patients with Type 2 Diabetes: A Systematic Review and Meta-Analysis of Randomized Controlled Trials

**DOI:** 10.1155/2021/5644171

**Published:** 2021-08-24

**Authors:** Tianqing Zhang, Qi He, Yao Liu, Zhenrong Chen, Hengjing Hu

**Affiliations:** ^1^The First Affiliated Hospital, Department of Cardiovascular Medicine, Hengyang Medical School, University of South China, Hengyang, Hunan Province, China; ^2^People's Hospital of Ningxiang City, Ningxiang City, Hunan Province, China; ^3^Institute of Cardiovascular Disease and Key Lab for Arteriosclerology of Hunan Province, University of South China, Hengyang, Hunan, China

## Abstract

**Background:**

Diabetes is a major public health concern. Resveratrol has shown great beneficial effects on hyperglycemia and insulin resistance and as an antioxidant.

**Methods:**

We searched the Chinese and English databases (such as CNKI, PubMed, and Embase) and extracted data from randomized controlled trials (RCTs). Then, RevMan 5.3 was used for bias risk assessment and meta-analysis. The primary outcome indicators include insulin-resistance-related indicators and blood-lipid-related indicators. This systematic review and meta-analysis was registered in PROSPERO (CRD42018089521).

**Results:**

Fifteen RCTs involving 896 patients were included. For insulin-resistance-related indicators, the summary results showed that, compared with the control group, homeostasis model assessment for insulin resistance (HOMA-IR) in the resveratrol group is lower (WMD: −0.99; 95% CI −1.61, −0.38; *P*=0.002). For blood-lipid-related indicators, the total cholesterol (TC) and triglyceride (TG) in the resveratrol group is of no statistical significance (for TC, WMD: −7.11; 95% CI −16.28, 2.06; *P*=0.13; for TG, WMD: −2.15; 95% CI −5.52, 1.22; *P*=0.21). For adverse events, the summary results showed that there was no statistical difference in the incidence of adverse events between the resveratrol and control groups (WMD: 2; 95% CI 0.44, 9.03; *P*=0.37).

**Conclusion:**

Based on the current evidence, resveratrol may improve insulin resistance, lower fasting blood glucose and insulin levels, and improve oxidative stress in patients with type 2 diabetes mellitus.

## 1. Introduction

Diabetes is a serious metabolic disease that affects about 5% of the world's people. Epidemiological data show that the number of people with diabetes is expected to increase dramatically to 592 million by 2035 [1]. 12% of global health expenditure is spent annually on diabetes and its complications [2]. Diabetes is divided into different types: type 1 and type 2 diabetes account for more than 90% of all cases. Metabolic abnormalities and serious complications caused by type 2 diabetes have profound effects on the life and quality of life of patients, such as microvascular (retinopathy, nephropathy), large blood vessels and peripheral vascular disease [3, 4], and increased risk of cancer [5, 6]. Type 2 diabetes mellitus (T2DM) is characterized by insulin resistance and hyperglycemia [7]. The treatment drugs for T2DM include insulin, alpha glucosidase inhibitors, dipeptidyl peptidase 4 inhibitors, incretin analogues, biguanides, insulin secretagogues, insulin sensitizers, and intestinal lipase inhibitors [8, 9]. However, the currently used therapies are accompanied by side effects, such as hypoglycemia, gastrointestinal problems, and weight gain [8]. Therefore, new drugs and natural compounds are constantly being tested to better prevent and treat diabetes [10].

In the alternative treatment strategy for diabetes treatment, resveratrol, a naturally occurring polyphenolic compound, mainly derived from the rhizome of the main natural source of *Polygonum cuspidatum*. Studies have shown that resveratrol has shown great beneficial effects on hyperglycemia, insulin resistance, and antioxidant [11, 12]. Clinical trials have shown that resveratrol has potential benefits for patients with T2DM, and relevant systematic reviews and reviews have also made relevant comments. However, some results contradict the evidence for the beneficial effects of resveratrol in the treatment of T2DM [13, 14]. This may be due to the limitation of sample size and treatment duration masking clear changes in clinical practice [12–14]. Meanwhile, the most recent meta-analysis search deadline was in June 2017, and a large number of RCTs appeared in the following period [15–20]. Therefore, we conduct a new systematic review and meta-analysis on this topic to evaluate the effects of resveratrol supplements on blood sugar, blood lipids, oxidative stress, safety, and other aspects of T2DM.

## 2. Materials and Methods

### 2.1. Protocol

This systematic review and meta-analysis were conducted strictly in accordance with the protocol (CRD42018089521) and PRISMA 2020 guidelines (see Supplementary Materials).

### 2.2. Inclusion and Exclusion Criteria

#### 2.2.1. Participants

Participants are patients with T2DM diagnosed through recognized standards, regardless of age, gender, and nationality. Records need to mention clear diagnostic criteria for RA, with a balanced baseline and comparability.

#### 2.2.2. Intervention

The intervention in experiments group was resveratrol supplements with no limits on the type, dose, frequency, and so on. The intervention in control group was western medicine, blanks, or placebo.

#### 2.2.3. Outcomes

The primary outcomes are as follows: homeostasis model assessment for insulin resistance (HOMA-IR), total cholesterol (TC), triglyceride (TG). The Secondary outcomes are as follows: HbA1c, low density lipoprotein cholesterol (LDL-C), high density lipoprotein cholesterol (HDL-C), fasting glucose, fasting insulin, and MDA.

#### 2.2.4. Study Type

This study is a randomized controlled trial (RCT), with no limits on the manner by which randomization has been achieved, blinding, or language of publication.

#### 2.2.5. Exclusion Criteria

The exclusion criteria are as follows: (1) not T2DM patients; (2) the participant is not human; (3) nonoriginal research literature; (4) non-RCT.

### 2.3. Search Strategy

The English databases (Web of Science, EMBASE, PubMed, and Medline Complete) and Chinese databases (China National Knowledge Infrastructure Databases (CNKI), Chinese Biomedical Database (CBM), Chinese Science and Technology Periodical Database (VIP), and Wan Fang Database) were searched. The search time period is from the establishment of the database to 16th of February, 2020. In addition, the Cochrane Library (until Issue 2, 2020) and clinical trial registration database (ClinicalTrials) were also searched. The search strategy for PubMed is presented in Table S1, as an example.

### 2.4. Literature Screening and Study Quality Assessment

Literature screening and study quality assessment were performed according to the Cochrane system evaluation method. First, the reviewers read the title and abstract for a preliminary screening and then screened them based on the full text. If there is a disagreement, it is resolved through discussion with all researchers. The lack of information would be supplemented by contacting the author through a letter or by imputation [21].

The quality of the literature was evaluated using the Cochrane bias risk assessment tool provided by the Cochrane Collaboration [22], and the following were evaluated: (1) whether the random method is correct; (2) whether the allocation is hidden; (3) blind method; (4) data bias; (5) selective reporting bias; (6) other biases. The evaluation was first conducted independently by two researchers. If there is a disagreement, it is resolved through discussion with all researchers.

### 2.5. Statistical Analysis

The RevMan version 5.3 statistical software provided by Cochrane Collaboration was used for analysis [23]. When the heterogeneity of RCTs was small (*P* > 0.1, *I*^2^ < 50%), the fixed-effects model was used for meta-analysis. If there is statistical heterogeneity (*P* < 0.1, *I*^2^ > 50%), the reviewers would first look for the source of heterogeneity. If the heterogeneity between the RCTs was statistical rather than clinical heterogeneity, the random-effects model would be used for meta-analysis. If the heterogeneity was too large or the data source cannot be found, a descriptive analysis would be performed. For continuous variables, the weighted mean difference (WMD) was used as the effect analysis statistic, and the interval is estimated using a 95% confidence interval (95% CI). If the difference in the value of the outcome exceeds 10 times or the unit of measurement is different, the standard MD (SMD) was used. For dichotomous variable, the risk ratio (RR) was used as the effect analysis statistic with 95% CI.

### 2.6. Sensitivity Analysis

STATA 15.0 was utilized for sensitivity analysis. The outcomes that met the following conditions were all subjected to sensitivity analysis: (1) random-effects model is used; (2) the results of the fixed-effects model are inconsistent with the results of the random-effects model (whether it is a subgroup result or a summary result).

## 3. Results

### 3.1. Results of the Search and Description of Included Trials

**Figure 1:**
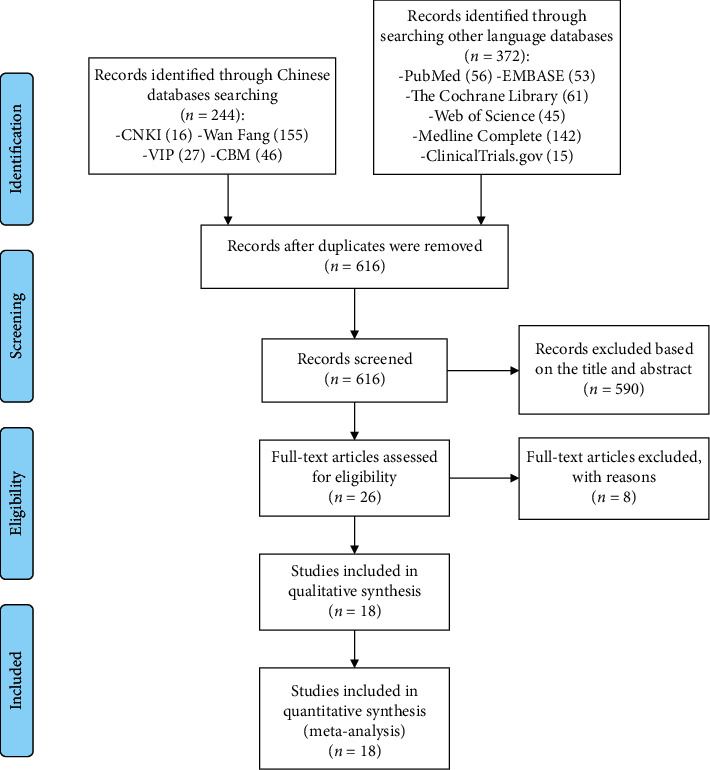
Flow diagram of searching and article selection.

A total of 616 articles were retrieved through the database: 590 articles were excluded by reading titles and abstracts, and eight articles were excluded by reading the full text. Finally, 18 articles met the inclusion criteria [15–19, 24–36] (Figure 1). Three studies are by Bo et al. [16, 29, 30], two studies are by Imamura et al. [17], and two studies are by Abdollahi et al. [33, 34], and they were merged. Study characteristics are presented in Table 1.

### 3.2. Risk of Bias Assessments

**Figure 2:**
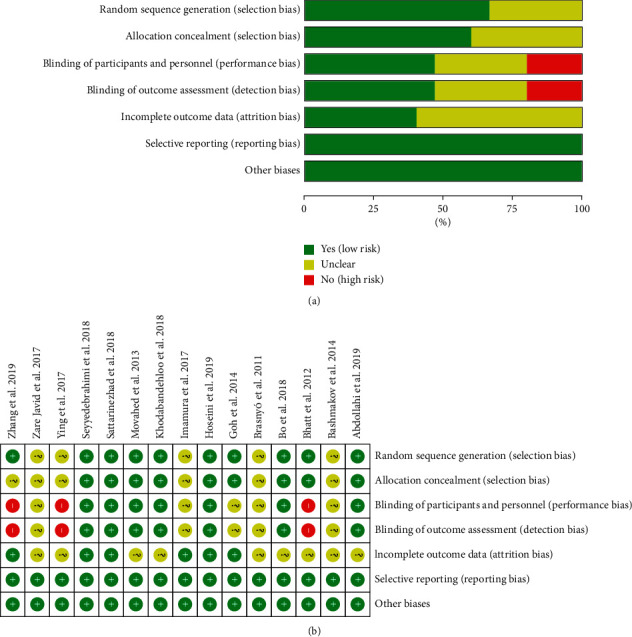
The risk of bias assessment. (a) The risk of bias graph; (b) the risk of bias table.

The summary and graph of risk of bias are shown in Figure 2.

#### 3.2.1. Random Sequence Generation and Allocation Concealment

Five RCTs [15, 17, 26, 27, 36] did not describe the method of generating random sequences and were rated as unclear risk of bias. Other RCTs describe the random sequence generation methods and are assessed as low risk of bias.

Six RCTs [15, 17, 26, 27, 35, 36] did not describe the allocation concealment method and were rated as having an unclear risk of bias. The other RCTs utilized the capsules in the same shape, size, and color to contain curcumin and placebo; hence, they were considered to have allocation concealment and rated as having low risks of bias.

#### 3.2.2. Blinding

Three RCTs [28, 35, 36] did not specify whether blinding was used and therefore were assessed as high risk of bias. The other RCTs claimed to use blinding, but Goh et al. [25], Brasnyó et al. [26], Bashmakov et al. [27], Zare Javid et al. [15], and Imamura et al. [17] did not describe the implementation process for both researchers and participants. They were rated as unclear risk of bias. The other RCTs described blinding of participants, so the blinding of participants and personnel (performance bias) was rated as low risk of bias.

#### 3.2.3. Incomplete Outcome Data and Selective Reporting

Six RCTs [17, 18, 25, 31, 32, 35] were assessed as unclear risk of bias because of missing data and did not describe whether to use intend-to-treat analysis. The incomplete outcome data of the other RCTs are rated as low risk of bias because the number of missing people and the reasons for the missing between groups is balanced. All RCTs reported study's prespecified outcomes that are of interest in the review; their risks of bias were low.

#### 3.2.4. Other Potential Bias

There were other sources of bias in all RCTs; therefore, the risks of other bias were low.

### 3.3. Primary Outcomes

#### 3.3.1. Homeostasis Model Assessment for Insulin Resistance

**Figure 3:**
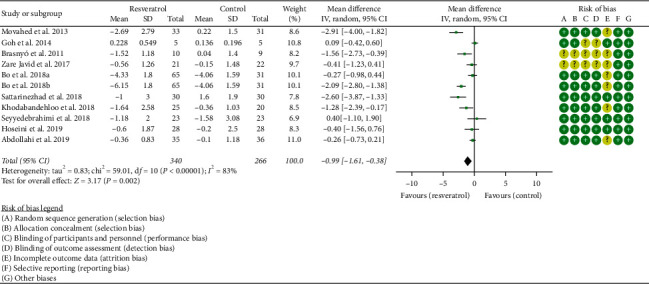
Homeostasis model assessment for insulin resistance (HOMA-IR).

Ten RCTs reported the changes in HOMA-IR, and there was a large statistical heterogeneity among the studies (*P* < 0.00001, *I*^2^ = 83%), so the random-effects model was used. The HOMA-IR of the resveratrol group was significantly lower than that of the control group, and the difference was statistically significant (WMD = −0.99; 95% CI (−1.61, −0.38); *P*=0.002; random-effect model) (Figure 3).

#### 3.3.2. Total Cholesterol

**Figure 4:**
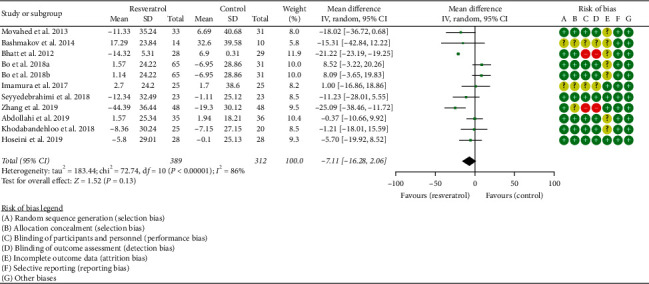
Total cholesterol.

Ten RCTs reported the changes in TC, and there was a large statistical heterogeneity among the studies (*P* < 0.00001, *I*^2^ = 86%), so the random-effects model was used. The results showed that there was no statistical difference in TC between the resveratrol and control groups (WMD = −7.11; 95% CI (−16.28, 2.06); *P*=0.13; random-effect model) (Figure 4).

#### 3.3.3. Triglyceride

**Figure 5:**
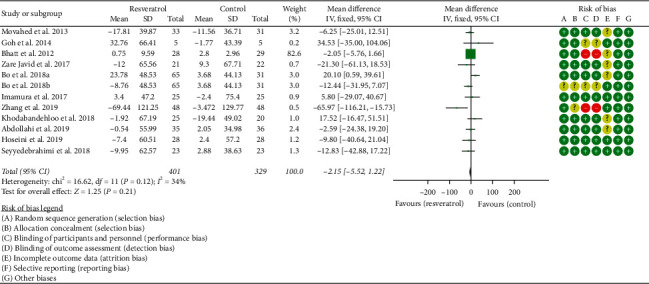
Triglyceride.

Eleven RCTs reported the changes in TG, and the statistical heterogeneity among the studies was low (*P*=0.12, *I*^2^ = 34%), so the fixed-effects model was used. The results showed that there was no statistical difference in TG between the resveratrol and control groups (WMD = −2.15; 95% CI (−5.52, 1.22); *P*=0.21; fixed-effect model) (Figure 5).

### 3.4. Secondary Outcomes

#### 3.4.1. Glycosylated Hemoglobin

**Figure 6:**
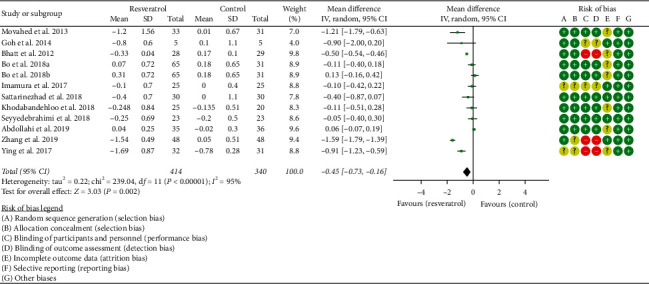
Glycosylated hemoglobin.

Eleven RCTs reported the changes in HbA1c, and there was a large statistical heterogeneity among the studies (*P* < 0.00001, *I*^2^ = 95%), so the random-effects model was used. The HbA1c of the resveratrol group was significantly lower than that of the control group, and the difference was statistically significant (WMD = −0.45; 95% CI (−0.73, −0.16); *P*=0.002; random-effect model) (Figure 6).

#### 3.4.2. Fasting Glucose and Fasting Insulin

**Figure 7:**
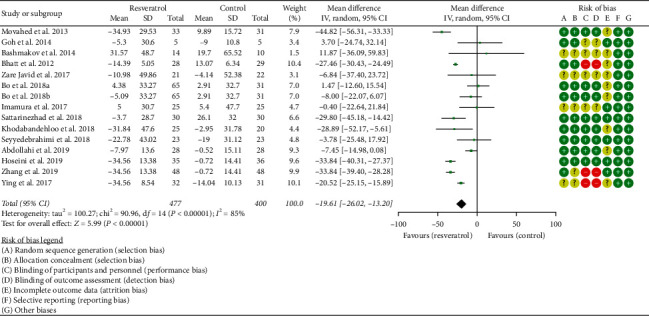
Fasting blood glucose.

Fourteen RCTs reported the changes in fasting glucose, and there was a large statistical heterogeneity among the studies (*P* < 0.00001, *I*^2^ = 85%), so the random-effects model was used. The fasting glucose of the resveratrol group was significantly lower than that of the control group, and the difference was statistically significant (WMD = −19.61; 95% CI (−26.02, −13.20); *P* < 0.00001; random-effect model) (Figure 7).

**Figure 8:**
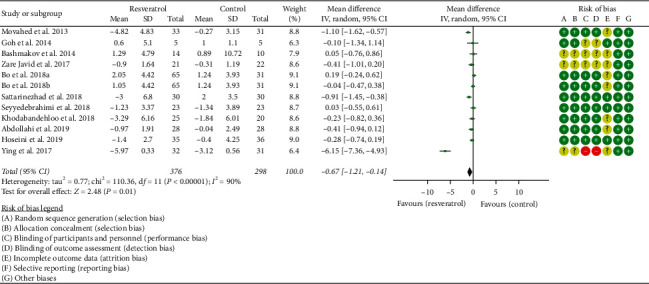
Fasting insulin.

Thirteen RCTs reported the changes in fasting insulin, and there was a large statistical heterogeneity among the studies (*P* < 0.00001, *I*^2^ = 90%), so the random-effects model was used. The fasting insulin of the resveratrol group was significantly lower than that of the control group, and the difference was statistically significant (SMD = −0.67; 95% CI (1.21, −0.14); *P*=0.01; random-effect model) (Figure 8).

#### 3.4.3. LDL-C and HDL-C

**Figure 9:**
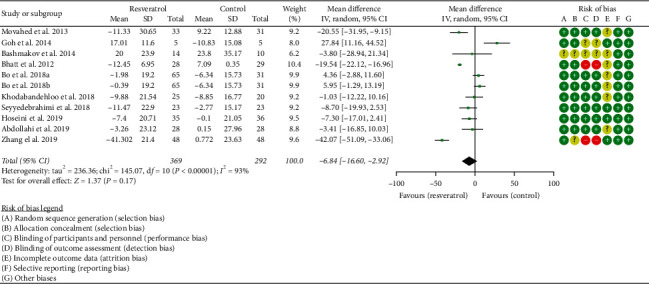
LDL-C.

Ten RCTs reported the changes in LDL-C, and there was a large statistical heterogeneity among the studies (*P* < 0.00001, *I*^2^ = 93%), so the random-effects model was used. The results showed that there was no statistical difference in LDL-C between the resveratrol and control groups (WMD = −6.84; 95% CI (−16.60, 2.92); *P*=0.17; random-effect model) (Figure 9).

**Figure 10:**
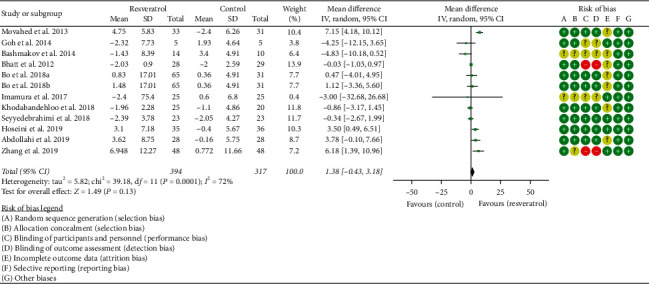
HDL-C.

Eleven RCTs reported the changes in HDL-C, and there was a large statistical heterogeneity among the studies (*P* < 0.0001, *I*^2^ = 72%), so the random-effects model was used. The results showed that there was no statistical difference in HDL-C between the resveratrol and control groups (WMD = 1.38; 95% CI (−0.43, 3.18); *P*=0.13; random-effect model) (Figure 10).

#### 3.4.4. Oxidative-Stress-Related Indicators

**Figure 11:**
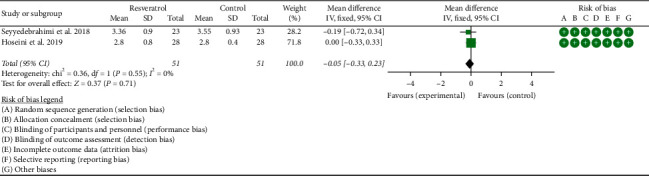
MDA.

Two RCTs reported the changes in MDA, and the statistical heterogeneity among the studies was low (*P*=0.55, *I*^2^ = 0%), so the fixed-effects model was used. The results showed that there was no statistical difference in MDA between the resveratrol and control groups (WMD = −0.05; 95% CI (−0.33, 0.23); *P*=0.71; fixed-effect model) (Figure 11).

### 3.5. Adverse Events

**Figure 12:**
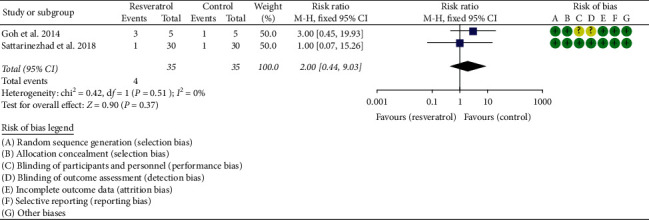
Adverse events.

Two RCTs reported the adverse events, and the statistical heterogeneity among the studies was low (*P*=0.51, *I*^2^ = 0%), so the fixed-effects model was used. The results showed that there was no statistical difference of adverse events between the resveratrol and control groups (RR = 2; 95% CI (0.44, 9.03); *P*=0.37; fixed-effect model)] (Figure 12).

### 3.6. Sensitivity Analysis Results

**Figure 13:**
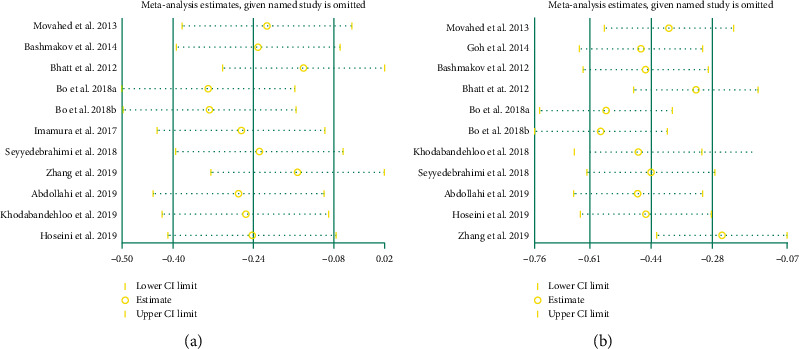
Sensitivity analysis results: (a) TC; (b) LDL-C.

Sensitivity analyses were performed for five outcomes: TC and LDL-C. (1) In the outcome “TC,” no matter which study was removed, the results were not significantly changed, suggesting that the heterogeneity may not come from RCT (Figure 13(a)). (2) In the outcome “LDL-C,” after we omitted the study of Zhang et al. [35], we found that the estimate of the result moved out of the lower limit of 95% CI (Figure 13(b)). This indicates that the study of Zhang et al. [35] may be the source of heterogeneity of LDL-C outcomes.

## 4. Discussion

This systematic review and meta-analysis included 15 RCTs involving 896 patients. This research showed that resveratrol may improve HOMA-IR and reduce HbA1c, fasting blood sugar, and fasting insulin levels, indicating that resveratrol may reduce insulin resistance, thereby lowering blood sugar and insulin levels. Although the results found in the current research are meaningful, they should be interpreted with caution due to the high heterogeneity of these results and small number of participants involved. This study did not show the positive effects of resveratrol on blood lipid levels and oxidative stress levels but showed that they have a trend of improvement. In the future, more RCTs may be needed to confirm or modify the effects of resveratrol on blood lipids and oxidative stress indicators in patients with T2DM. Only two RCTs reported adverse events, and the meta-analysis results showed that there was no statistically significant difference in adverse events between the control and resveratrol groups. Due to the insufficient number of RCTs, this result is doubtful. It can only be inferred based on the existing evidence that resveratrol may be a safe therapy for the treatment of T2DM. More RCTs are needed in the future to report on the safety of resveratrol.

Resveratrol, as a type of polyphenolic phytoalexin, has good antioxidant properties. It is produced by plants under the action of exogenous stimuli, such as ultraviolet light irradiation, mechanical damage or fungal infection [37–41]. A large number of in vitro and in vivo tests have shown that resveratrol can effectively prevent hypertension through antioxidant effects [42], cardiovascular diseases [43], nonalcoholic fatty liver [44], metabolic syndrome [45], aging [46], cancer [47], and immunological diseases [48], through its antioxidant effect, and has a good application prospect. Based on this, the research on the safety of resveratrol is meaningful. Williams et al. [49] showed that resveratrol is not irritating to the skin and eyes, and the micronucleus test in vivo proved that resveratrol has no genetic toxicity. After a 90-day subchronic toxicity test, it was found that resveratrol did not cause any adverse effects on the body and did not have reproductive toxicity at the maximum dose of 700 mg/(kg·d). This preliminarily proves that resveratrol is nontoxic and safe. Hebbar et al. [50] administered resveratrol to CD rats at 0.3, 1.0, and 3.0 g/(kg·d). They found that, at 0.3 g/(kg·d), the rats showed no adverse reactions. However, at 1.0 and 3.0 g/(kg·d), female and male rats experienced different degrees of dehydration, dyspnea, kidney toxicity, and increased serum liver enzymes. It shows that resveratrol has certain toxicity at high doses. In order to determine the safe dose range of resveratrol, Johnson et al. [51] also studied the subchronic oral toxicity of resveratrol. The results showed that when the dose was increased to 1 000 mg/(kg·d), resveratrol showed certain toxicity; it showed that the no-observed-adverse-effect levels (NOAELs) of resveratrol in rats and dogs are 200 mg/(kg·d) and 600 mg/(kg·d), respectively. Since the content of resveratrol in plants or foods is lower than NOAEL, it can be considered that normal consumption of foods rich in resveratrol can not only give full play to its physiological activities but also be safe. In clinical trials, a randomized, double-blind, placebo-controlled clinical trial found that, at a clinical dose of 150 mg/day, no effect of resveratrol supplementation on cardiometabolic risk parameters was observed. It suggests that resveratrol supplements are well tolerated and safe [52]. Federica et al. found that high daily doses (≥300 mg/day) of resveratrol can promote cardiovascular health. Resveratrol is well tolerated, and no serious adverse events occurred in most eligible trials [53]. This study also showed that the adverse events of the resveratrol group were the same as those of the control group, and no serious adverse events occurred, which suggested that resveratrol has good safety.

Most of the results, such as HOMA-IR, TC, TG, and LDL-C, have large heterogeneity, so this study used sensitivity analysis to find the source of heterogeneity. Sensitivity analyses were performed for TC and LDL-C. For outcome “LDL-C,” the study of Zhang et al. [35] may be the source of heterogeneity. Compared with other studies, the blinding method of Zhang et al. [35] was assessed as a high risk of bias, and allocation concealment was assessed as an unknown risk of bias. This suggests that the heterogeneity may be due to the low quality of RCTs, and failure to apply blinding may lead to biased results. In addition to the possible sources of heterogeneity found by sensitivity analysis, heterogeneity may also come from ethnic differences, regional differences, gender differences, and so on: (1) most of the RCTs are from Iran, and a few are from China, Japan, Egypt, Singapore, Hungary, and Italy (see Table 1); there are differences between races in these countries, and this may cause different sensitivities to resveratrol. (2) The gender composition ratio of RCTs is different. The participants in the studies of Goh et al. [25] and Brasnyó et al. [26] were all male, while the gender ratio in the study of Hoseini et al. [32] is not clear. Males and females having different sensitivities to drugs may lead to heterogeneity. (3) The dosage, preparation type, and usage of resveratrol in RCTs are different. The difference between the dosage and type of preparation may affect the efficacy of the drug, which may be the source of heterogeneity.

This meta-analysis is similar to the works of Zhu et al. [13] and Liu et al. [14] in that both have shown that resveratrol can improve HOMA-IR, fasting blood glucose, and HbA1c insulin levels. The differences are as follows. (1) *Research Process.* This research was registered with PROSPERO in advance, and it was analyzed strictly according to the protocol and PRISMA-guidelines. The inclusion and exclusion criteria were more stringent. (2) *Literature Quality Assessment.* Cochrane bias risk assessment tool is used to evaluate the literature quality. (3) *Included Literature.* Liu et al. [14] only evaluated RCTs of types 1 and 2 diabetes, while Zhu et al. [13] only evaluated nine RCTs. This study evaluated 15 diabetes-related RCTs; 10 of them [15–19, 29–36] are published after 2016, which showed more stable results.

The advantage of this study is that the systematic review and meta-analysis include all available RCTs for clinical problems. The study is the latest systematic review and meta-analysis on this topic, and it is conducted in strict accordance with the guidelines and protocol. In addition, this study included a wider range of populations, including Iran, China, Japan, Egypt, Singapore, Hungary, and Italy, which promoted the applicability of the conclusions. The limitations of this study are as follows: (1) some RCTs are of low quality because they did not describe specific random sequence generation methods, allocation concealment methods, or blind methods, which lead to a decrease in the reliability of the results, and the results should be treated with caution in clinical practice. For example, five RCTs [15, 17, 26, 27, 36] did not describe the method of generating random sequences; six RCTs [15, 17, 26, 27, 35, 36] did not describe the allocation concealment method; three RCTs [28, 35, 36] did not specify whether blinding; six RCTs [17, 18, 25, 31, 32, 35] did not describe whether to use intend-to-treat analysis. (2) Some RCTs involve few participants (lower than 50), which may lead to changes in clinical efficacy indicators that cannot be detected. Goh et al. [25] included only 10 participants; Brasnyó et al. [26], 19 participants; Bashmakov et al. [27], 24 participants; Zare Javid et al. [15], 43 participants; Khodabandehloo et al. [19], 45 participants; Seyyedebrahimi et al. [31], 46 participants. (3) The different resveratrol preparations used by RCTs may affect the accuracy of the results. For example, the dose and usage of resveratrol in the study of Brasnyó et al. [26] was 5 mg, bid; in the study of Bashmakov et al. [27], 50 mg, bid; in the study of Movahed et al. [24], 500 mg, bid. The dosage of resveratrol in each RCT was different. (4) The heterogeneity of most outcomes, such as HOMA-IR, TC, TG, and LDL-C, was high. The high heterogeneity may reduce the applicability of the results. Therefore, high-quality research is needed to determine or modify the results of this research.

## 5. Conclusion

Resveratrol may improve insulin resistance, lower fasting blood glucose and insulin levels, and improve oxidative stress in patients with T2DM. However, due to the generally low quality of research and high heterogeneity among RCTs, the results should be interpreted with caution.

## Figures and Tables

**Table 1:** The characteristics of the included studies.

Study	Country	Sample size (female/male)		Intervention		Relevant outcomes	Mean age (years)		Duration
		Trial group	Control group	Trial group	Control group		Trial group	Control group	
Movahed et al. [23]	Iran	33 (16/17)	31 (17/16)	Resveratrol 500 mg, bid	Microcellulose (placebo) 500 mg, bid	HOMA-IR, HbA1c, fasting blood glucose, fasting insulin, TC, TG, HDL-C, LDL-C	52.45 ± 6.18	51.81 ± 6.99	1.5 months
Goh et al. [24]	Singapore	5 (0/5)	5 (0/5)	Resveratrol 500 mg, qd, initially, increased by 500 mg per day every 3 days, to a maximum dose of 3000 mg per day (1000 mg, tid)	Placebo 500 mg, qd, initially, increased by 500 mg per day every 3 days, to a maximum dose of 3000 mg per day (1000 mg, tid)	HbA1c, fasting blood glucose, fasting insulin, TC, TG, HDL-C, LDL-C, adverse events	55.8 ± 7.3	56.8 ± 5.3	3 months

Brasnyó et al. [25]	Hungary	10 (0/10)	9 (0/9)	Resveratrol 5 mg, bid	Placebo, bid	HOMA-IR	57.79 ± 7.9	52.5 ± 11.1	1 month

Bashmakov et al. [26]	Egypt	14 (6/8)	10 (3/10)	Resveratrol 50 mg, bid	Placebo, bid	Fasting blood glucose, fasting insulin, TC, HDL-C, LDL-C	54.0 ± 10.1	59.8 ± 6.6	2 months

Bhatt et al. [27]	India	28 (20/9)	29 (16/12)	Resveratrol 250 mg, qd, with oral hypoglycemic agents such as glibenclamide and/or metformin	Oral hypoglycemic agents such as glibenclamide and/or metformin	HbA1c, fasting blood glucose, TC, TG, HDL-C, LDL-C	56.67 ± 8.91	57.75 ± 8.71	3 months

Zare Javid et al. [15]	Iran	21 (18/4)	22 (16/5)	Resveratrol 240 mg, bid	Starch (placebo) 240 mg, bid	HOMA-IR, fasting blood glucose, fasting insulin, TG	49.1 ± 7.4	50.9 ± 8.9	1 month

Bo et al. [16, 28, 29]	Italy	130 (51/79)	62 (15/47)	Resveratrol 500 mg, qd, or 40 mg, qd	Placebo, qd	HOMA-IR, HbA1c, fasting blood glucose, fasting insulin, TC, TG, HDL-C, LDL-C	64.95 ± 8.08	65.4 ± 8.8	6 months

Imamura et al. [17]	Japan	25 (10/15)	25 (14/11)	Resveratrol 100 mg, qd	Placebo, qd	HbA1c, fasting blood glucose, fasting insulin, TC, TG, HDL-C, adverse events	57.4 ± 10.6	58.2 ± 10.1	3 months
Sattarinezhad et al. [18]	Iran	30 (16/14)	30 (17/13)	Resveratrol 500 mg, qd + losartan 12.5 mg, qd	Placebo 500 mg, qd + losartan 12.5 mg, qd	HOMA-IR, HbA1c, fasting blood glucose, fasting insulin, adverse events	56.8 ± 9.7	55.7 ± 10.8	3 months

Khodabandehloo et al. [19]	Iran	25 (12/13)	20 (10/10)	Resveratrol 400 mg, bid	Microcellulose (placebo) 400 mg, bid	HOMA-IR, HbA1c, fasting blood glucose, fasting insulin, TC, TG, HDL-C, LDL-C, adverse events	56.48 ± 6.72	61.10 ± 5.61	2 months

Seyyedebrahimi et al. [30]	Iran	23 (12/11)	23 (13/10)	Resveratrol 400 mg, bid	Microcellulose (placebo) 400 mg, bid	HOMA-IR, HbA1c, fasting blood glucose, fasting insulin, TC, TG, HDL-C, LDL-C, MDA, adverse events	54.96 ± 6.37	58.72 ± 6.06	2 months

Hoseini et al. [31]	Iran	28 (unknown/unknown)	28 (unknown/unknown)	Resveratrol 500 mg, qd	Placebo, qd	HOMA-IR, fasting blood glucose, fasting insulin, TG, TC, HDL-C, LDL-C, MDA, TAC, adverse events	61.0 ± 8.6	63.3 ± 10.1	1 month
Abdollahi et al. [32, 33]	Iran	35 (15/20)	36 (16/20))	Resveratrol 500 mg, bid	Placebo, bid	HOMA-IR, HbA1c, fasting blood glucose, fasting insulin, TG, TC, HDL-C, LDL-C, adverse events	50.14 ± 7.38	50.06 ± 7.69	2 months

Zhang et al. [34]	China	48 (24/24)	48 (23/25)	Resveratrol 300 mg, bid	Blank	HbA1c, fasting blood glucose, fasting insulin, TG, TC, HDL-C, LDL-C	50.9 ± 9.7	52.3 ± 11.2	3 months

Ying et al. [35]	China	32 (12/20)	31 (11/20)	Resveratrol 500 mg, bid	Blank	HbA1c, fasting blood glucose, fasting insulin	64.94 ± 1.36	64.95 ± 1.35	2 months

HOMA-IR: homeostasis model assessment for insulin resistance; TC: total cholesterol; TG: triglyceride; LDL-C: low density lipoprotein cholesterol; HDL-C: high density lipoprotein cholesterol.

## Data Availability

All data generated or analyzed during this study are included within the article.

## Supplementary Materials

Supplementary Materials The “PRISMA 2020 checklist” is a standardized checklist that ensures that systematic reviews and meta-analyses are implemented according to this standard. Table S1: search strategies for PubMed.
