# Supplementary material for: Efficacy and Safety of Resveratrol Supplements on Blood Lipid and Blood Glucose Control in Patients with Type 2 Diabetes: A Systematic Review and Meta-Analysis of Randomized Controlled Trials
Source: Evid Based Complement Alternat Med. 2021 Aug 24;2021:5644171. doi: 10.1155/2021/5644171 (PMC8410426; doi:10.1155/2021/5644171)
Supplement: Supplementary Materials — The “PRISMA 2020 checklist” is a standardized checklist that ensures that systematic reviews and meta-analyses are implemented according to this standard. Table S1: search strategies for PubMed. [file 5644171.f1.zip › 5644171.f1/Table S1. Search Strategies for Pubmed.pdf]

Table S1. Search Strategies for Pubmed

**PubMed**	(resveratrol OR resveratrols OR 3,4',5-stilbenetriol OR 3,5,4'-trihydroxystilbene OR trans-resveratrol-3-O-sulfate OR SRT 501 OR SRT501 OR SRT-501 OR trans-resveratrol OR cis-resveratrol OR resveratrol-3-sulfate)<br>AND<br>(Type 2 diabetes mellitus OR Diabetes Mellitus, Noninsulin-Dependent OR Diabetes Mellitus, Ketosis-Resistant OR Diabetes Mellitus, Ketosis Resistant OR Ketosis-Resistant Diabetes Mellitus OR Diabetes Mellitus, Non Insulin Dependent OR Diabetes Mellitus, Non-Insulin-Dependent OR Non-Insulin-Dependent Diabetes Mellitus OR Diabetes Mellitus, Stable OR Stable Diabetes Mellitus OR Diabetes Mellitus, Type II OR NIDDM OR Diabetes Mellitus, Noninsulin Dependent OR Diabetes Mellitus, Maturity-Onset OR Diabetes Mellitus, Maturity Onset OR Maturity-Onset Diabetes Mellitus OR Maturity Onset Diabetes Mellitus OR MODY OR Diabetes Mellitus, Slow-Onset OR Diabetes Mellitus, Slow Onset OR Slow-Onset Diabetes Mellitus OR Type 2 Diabetes Mellitus OR Noninsulin-Dependent Diabetes Mellitus OR Noninsulin Dependent Diabetes Mellitus OR Maturity-Onset Diabetes OR Diabetes, Maturity-Onset OR Maturity Onset Diabetes OR Type 2 Diabetes OR Diabetes, Type 2 OR Diabetes Mellitus, Adult-Onset OR Adult-Onset Diabetes Mellitus OR Diabetes Mellitus, Adult Onset)<br>AND<br>(randomized controlled trial [pt] OR controlled clinical trial [pt] OR trial [tiab] OR clinical trials as topic [mesh: noexp] OR Clinical Trial OR random* [tiab] OR random allocation [mh] OR single-blind method [mh] OR double-blind method [mh])<br>NOT<br>(animals [mh] NOT humans [mh])
